# Analysis of public opinion evolution of COVID-19 based on LDA-ARMA hybrid model

**DOI:** 10.1007/s40747-021-00514-7

**Published:** 2021-09-04

**Authors:** Muni Zhuang, Yong Li, Xu Tan, Lining Xing, Xin Lu

**Affiliations:** 1 School of Software Engineering, Shenzhen Institute of Information Technology, Shenzhen, 518172 China; 2 National Institute for Data Science in Health and Medicine, Xiamen University, Xiamen, 361005 China; 3 School of Economics and Management, Changsha University, Changsha, 410022 China; 4 College of Systems Engineering, National University of Defense Technology, Changsha, 410022 China

**Keywords:** Coronavirus disease 2019 (COVID-19), LDA-ARMA hybrid model, Large-scale online public opinion, Emotional evolution, Dynamic prediction

## Abstract

The aim of this study was to explore a method for developing an emotional evolution classification model for large-scale online public opinion of events such as Coronavirus Disease 2019 (COVID-19), in order to guide government departments to adopt differentiated forms of emergency management and to correctly guide online public opinion for severely afflicted areas such as Wuhan and those afflicted elsewhere in China. We propose the LDA-ARMA deep neural network for dynamic presentation and fine-grained categorization of a public opinion events. This was applied to a huge quantity of online public opinion texts in a complicated setting and integrated the proposed sentiment measurement algorithm. To begin, the Latent Dirichlet Allocation (LDA) was employed to extract information about the topic of comments. The autoregressive moving average model (ARMA) was then utilized to perform multidimensional sentiment analysis and evolution prediction on large-scale textual data related to COVID-19 published by netizens from Wuhan and other countries on Sina Weibo. The results show that Wuhan netizens paid more attention to the development of the situation, treatment measures, and policies related to COVID-19 than other issues, and were under greater emotional pressure, whereas netizens in the rest of the country paid more attention to the overall COVID-19 prevention and control, and were more positive and optimistic with the assistance of the government and NGOs. The average error in predicting public opinion sentiment was less than 5.64%, demonstrating that this approach may be effectively applied to the analysis of large-scale online public sentiment evolution.

## Introduction

At the moment, of public health incidents are unavoidable for our human society, most notably an outbreak of a novel coronavirus pneumonia (COVID-19) in Wuhan, China, in early 2020. This was a major public health emergency, with the highest speed of transmission, the broadest range of infection, and the highest level of difficulty of prevention and control since the founding of the People's Republic of China [1]. It was also listed by the World Health Organisation as the sixth most significant global public health emergency, after the Ebola epidemic in Africa in 2019 [2, 3]. Due to the large number of casualties and high economic and property losses, the physical and mental health of much of the population was threatened to a considerable extent, resulting in different degrees of tension, panic and other negative emotions [4]. In his work on the prevention of COVID-19, General Secretary Xi Jinping emphasised that “we must mobilise all kinds of forces to comprehensively strengthen psychological counselling” and has specifically pointed out that “we must strengthen the guidance of public opinion.” COVID-19 has produced massive amounts of public opinion data in the form of text on the Internet. In this context, it is quite necessary to analyse this valuable public sentiment information, clarify the development context, the characteristics and laws of public emotion and opinion evolution on COVID-19, explore the differences in emotional evolution between Wuhan and rest of China and provide specific measures for public opinion prevention and response to government departments. These content fall into the research category of big data text mining and public sentiment analysis.

Traditional sentiment analysis methods are mainly based on dictionaries, machine learning, Dictionary Plus Machine learning, weak labelling and deep learning [5]. For example, Rao et al. proposed a social sentiment monitoring dictionary based on a sentiment dictionary [6]; Tripathy et al. combined the naive Bayes (NB) approach, maximum entropy (ME), stochastic gradient descent (SGD) and support vector machine (SVM) machine learning algorithms with n-gram models for sentiment analysis [7]; Jie et al. proposed a classification algorithm that combined a dictionary with machine learning [8]; and Ma et al. proposed the enhancement of the long short-term memory (LSTM) artificial neural network by integrating both explicit and implicit knowledge, in an approach known as perceptual LSTM [9]. Experimental results have shown that these methods improved the accuracy of sentiment analysis to a certain extent.

However, these traditional methods of sentiment analysis are limited to judging the overall sentiment of the entire text, and rely on the manual labelling of text features, meaning that the processing efficiency is low when handling large amounts of unstructured text information. Topic extraction technology [5] is an emerging technology for the automatic annotation and extraction of words, phrases and sentences in the field of text mining, and can effectively solve this problem. At present, a great deal of research on topic extraction technology is applied in the fields of emotional information mining, emotional tendency analysis, emotional evolution analysis, and performance evaluation, such as the term frequency-inverse document frequency (TF-IDF) algorithm (based on word frequency statistics [10]), probabilistic latent semantic analysis (PLSA) [11] and latent Dirichlet allocation (LDA) [12] (based on the topic probabilistic model), automatic keyword extraction systems such as GenEx [13], KEA [14] (based on machine learning) and high-level text feature extraction and sentiment classification models (based on deep learning) [15].

It is worth noting that in recent years, LDA has been successfully utilized to classify the sentiments. He and Lin propose a novel probabilistic modelling approach for sentiment analysis based on LDA termed the joint sentiment/topic model (JSP) [16]. This model is completely unsupervised. Li et al. present a Sentiment-LDA model for sentiment analysis with global topics and local dependence [17]. Jo and Oh describe two models, Sentence-LDA (SLDA) and Aspect and Sentiment Unification Model (ASUM), to address the challenge of automatically determining which aspects are evaluated in reviews and how sentiments for different aspects are expressed [18]. Sentiment classification is done at a finer level, down to phrases and aspects. However, sentiment information is not used in these works during modelling.

In the field of sentiment prediction, the Box-Jenkins methodology, which employs autoregressive moving average model (ARMA) linear models, has dominated several fields of time series forecasting. Box and Jenkins [19] popularized ARMA models in 1970 by developing a model-building methodology that included an iterative three-stage process of model selection, parameter estimation, and model checking.In a real-world scenario, text may be expressed in a complex context that includes English, slang, emoticons, etc. To obtain better results, we propose an improved LDA-ARMA theme analysis model that can effectively solve the problem of complex public opinion analysis.

First, we construct a Python crawler framework to capture content relating to COVID-19 from microblogs on Sina Weibo published by netizens of Wuhan and others across China. In this complex context, which includes the mixed use of Chinese and English and emoticons, we aimed to model the dynamic evolution and obtain a fine-grained understanding of the emotional evolution of public opinion of an event from a multi-dimensional perspective, and to predict and analyse the laws and emotional trends of this evolution. We consider that the weights of feature words in the original LDA “bag-of-words” models is uniform, which leads to the problem that the topic model tends to high-frequency words. We introduced a Gaussian function to set different weights for feature words in order to improve the rationality and independence of the distribution of topics. Then, based on the four mainstream sentiment dictionaries, we manually labelled specific sentiment words for the event and emoticons after sinicisation, and a sentiment value measurement algorithm based on machine learning was proposed that was combined with the ARMA model to give reasonable predictions of the emotional evolution of this epidemic event. We therefore explore the rules of evolution and the differences between netizens of Wuhan and others in terms of public opinion on COVID-19, and forms the whole process public opinion analysis framework from collection and processing, mining and analysis to strategy support. It provides certain theoretical basis support and scientific decision support for optimizing the public opinion collection function of microblog platform and assisting relevant management departments to effectively guide and control network public opinion when dealing with similar events.

The main contributions of our proposed work are highlighted as follows: (a) We introduced a Gaussian function to improve the LDA model. This method can achieve a more rational and fine-grained division of text topics; (b) we propose an emotional value measurement algorithm suitable for complex contexts; (c) we propose a large-scale network sentiment evolution model that combines improved LDA and ARMA models.

The remainder of the paper is organized as follows: Section “Sentiment analysis and prediction algorithm based on the hybrid LDA-ARMA model” improved the LDA topic model and ARMA time series model, and describes our proposed model and algorithm in detail; empirical analysis of public opinion and emotions in regard to COVID-19 are presented in Sect. “Empirical analysis of public opinion and emotions in regard to COVID-19”; Sect. “Analysis of the differences in the evolution of public opinion and sentiment between Netizens of Wuhan and other locations in China” analysis of the differences in the evolution of public opinion and sentiment between netizens of Wuhan and other locations in China; finally, Sect. “Conclusion” concludes this paper.

## Sentiment analysis and prediction algorithm based on the hybrid LDA-ARMA model

### Improved LDA topic model

**Fig. 1:**
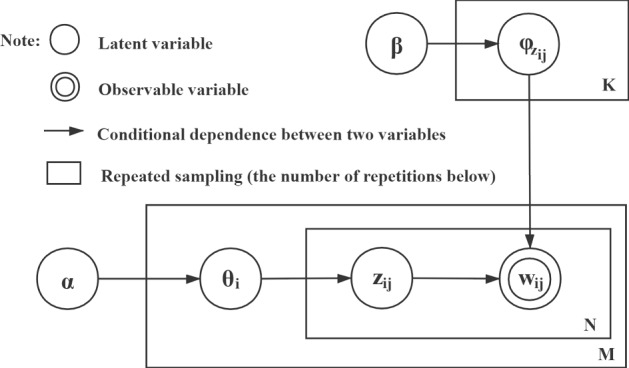
LDA topic analysis model

LDA is a document topic generation model that was proposed by Blei [12] in 2003. The model analyses the topics of text documents in the form of a probability distribution, and performs topic clustering or text classification according to this distribution. The modelling process is illustrated in Fig. 1. ${\varvec{D}}=\left\{{{\varvec{d}}}_{{\varvec{i}}}|{\varvec{i}}\in \{{1,2},\dots ,{\varvec{M}}\}\right\}\boldsymbol{ }$ is defined as a set containing M documents; ${{\varvec{d}}}_{{\varvec{i}}}=\left\{{{\varvec{d}}}_{{\varvec{i}}{\varvec{s}}}|{\varvec{s}}\in \left\{{1,2},\dots ,{\varvec{S}}\right\}\right\}$ is a document composed of S sentences (i.e. a set of sentences); ${{\varvec{w}}}_{{\varvec{i}}}=\{{{\varvec{w}}}_{{\varvec{i}}{\varvec{j}}}|\boldsymbol{ }{\varvec{j}}\in \{{1,2},\dots ,{{\varvec{N}}}_{{\varvec{i}}}\}\}$ is the set of words resulting from word segmentation of document ${{\varvec{d}}}_{{\varvec{i}}}$; ${{\varvec{N}}}_{{\varvec{i}}}$ is the number of words in document ${{\varvec{d}}}_{{\varvec{i}}}$; **N=**$\sum_{{\varvec{i}}=1}^{{\varvec{M}}}{{\varvec{N}}}_{{\varvec{i}}}$ is the number of words in the set of documents D; ${{\varvec{z}}}_{{\varvec{i}}}=\{{{\varvec{z}}}_{{\varvec{i}}{\varvec{j}}}|\boldsymbol{ }{\varvec{j}}\in \{{1,2},\dots ,{{\varvec{N}}}_{{\varvec{i}}}\}\}$ is the set of topics corresponding to the set of words ${{\varvec{w}}}_{{\varvec{i}}}$; and **K =|**$\bigcup_{{\varvec{i}}=1}^{{\varvec{M}}}{{\varvec{z}}}_{{\varvec{i}}}$***|*** is the total number of topics in the document set D. The LDA modelling process can be parsed as follows:

Step 1. Set the initial probability of each document ${{\varvec{d}}}_{{\varvec{i}}}$ to ${\varvec{P}}({{\varvec{d}}}_{{\varvec{i}}})$;

Step 2. Given the parameter ${\varvec{\upalpha}}$, which obeys the Dirichlet distribution, sample from the distribution to generate the topic distribution ${{\varvec{\theta}}}_{{\varvec{i}}}$ for document ${{\varvec{d}}}_{{\varvec{i}}}$;

Step 3. Sample from the topic distribution ${{\varvec{\theta}}}_{{\varvec{i}}}$ to generate the theme ${{\varvec{z}}}_{{\varvec{i}}{\varvec{j}}}$ of the jth word in the document ${{\varvec{d}}}_{{\varvec{i}}}$;

Step 4. Given the parameter **β** which obeys the Dirichlet prior distribution, sample from this distribution to generate the word distribution ${\boldsymbol{\varphi }}_{{{\varvec{z}}}_{{\varvec{i}}{\varvec{j}}}}$ of topic ${{\varvec{z}}}_{{\varvec{i}}{\varvec{j}}}$;

Step 5. Generate word ${{\varvec{w}}}_{{\varvec{i}}{\varvec{j}}}$ from the word distribution ${\boldsymbol{\varphi }}_{{{\varvec{z}}}_{{\varvec{i}}{\varvec{j}}}}$.

In the original LDA topic model, the text is expressed in the form of “bag-of-words”, which disregard the differences in weight. The distribution of topics is often inclined towards high-frequency words, which affects the accuracy. Therefore, inspired by Wang Rui's research [20], we use a Gaussian function-based word weight distribution to identify potential topic information from within a large-scale text. The ideas on which this improvement are based are as follows:

First, the Gaussian function shown in (1) is used to differentiate the words.

1 $$F({w}_{ij})=\frac{1}{\upsigma \sqrt{2\uppi }}\mathrm{exp}\left[-\frac{{({Fr}_{{w}_{ij}}-{Mn}_{{w}_{i}})}^{2}}{{2\sigma }^{2}}\right]$$

In this equation, ${{\varvec{F}}{\varvec{r}}}_{{{\varvec{w}}}_{{\varvec{i}}{\varvec{j}}}}$ is the word frequency of the word ${{\varvec{w}}}_{{\varvec{i}}{\varvec{j}}}$ in the document ${{\varvec{d}}}_{{\varvec{i}}}$, ${{\varvec{M}}{\varvec{n}}}_{{{\varvec{w}}}_{{\varvec{i}}}}$ is the average frequency of all words in the set ${{\varvec{w}}}_{{\varvec{i}}}$ corresponding to the document ${{\varvec{d}}}_{{\varvec{i}}}$, and ${{\varvec{\sigma}}}^{2}$ represents the variance.

Next, in order to ensure that the total number of words in the improved document ${{\varvec{d}}}_{{\varvec{i}}}$ remains unchanged, the weight of word ${{\varvec{w}}}_{{\varvec{i}}{\varvec{j}}}$ is calculated using (2):

2 $${\mathrm{Weight}}_{{w}_{ij}}=\frac{{N}_{i}\times F({w}_{ij})}{\sum_{j=1}^{{N}_{i}}{Fr}_{{w}_{ij}}\times F({w}_{ij})}$$

In order to better analyse the improved word weight ${\mathbf{W}\mathbf{e}\mathbf{i}\mathbf{g}\mathbf{h}\mathbf{t}}_{{{\varvec{w}}}_{{\varvec{i}}{\varvec{j}}}}$ calculation method, in the process of Gibbs sampling algorithm derivation of parameters ***θ*** and ***φ***, the joint distribution of all variables in the LDA model is calculated as shown in (3) as follows:

3 $$P\left({w}_{i},{z}_{i},{\theta }_{i},\Phi \left|\alpha \right.,\beta \right)=\prod_{j=1}^{{N}_{i}}P({w}_{ij}\left|{\varphi }_{{Z}_{ij}}\right.)P({z}_{ij}\left|{\theta }_{i})\cdot \right.P({\theta }_{i}\left|\alpha )\cdot \right.P(\Phi \left|\beta )\right.$$

In this equation, $\prod_{{\varvec{j}}=1}^{{{\varvec{N}}}_{{\varvec{i}}}}{\varvec{P}}({{\varvec{w}}}_{{\varvec{i}}{\varvec{j}}}\left|{\boldsymbol{\varphi }}_{{{\varvec{Z}}}_{{\varvec{i}}{\varvec{j}}}}\right.){\varvec{P}}({{\varvec{z}}}_{{\varvec{i}}{\varvec{j}}}\left|{{\varvec{\theta}}}_{{\varvec{i}}})\right.$ is the probability of all words in the ith document produced by ${{\varvec{\theta}}}_{{\varvec{i}}}$;${\varvec{P}}({{\varvec{\theta}}}_{{\varvec{i}}}\left|\boldsymbol{\alpha })\right.$ is the “text topic” distribution probability of the document ${{\varvec{d}}}_{{\varvec{i}}}$ generated by the Dirichlet parameter ***α*** defined above; and ${\varvec{P}}(\boldsymbol{\Phi }\left|{\varvec{\beta}})\right.$ is the “topic word” distribution matrix generated by the Dirichlet parameter ***β*** defined above.

Then according to (3), using Bayes’ rule and the Dirichlet distribution, a Dirichlet-multinomial conjugation is used to obtain Gibbs samples. The estimated values of parameters ${\boldsymbol{\varphi }}_{{{\varvec{z}}}_{{\varvec{i}}{\varvec{j}}}}^{{\varvec{c}}}$ and ${{\varvec{\theta}}}_{{{\varvec{z}}}_{{\varvec{i}}{\varvec{j}}}}^{{{\varvec{d}}}_{{\varvec{i}}}}$ in the Bayesian framework are expressed as in (4) and (5):

4 $${\widehat{\varphi }}_{{z}_{ij}}^{c}=\frac{{\mathrm{Num}}_{{z}_{ij}}^{c}+{\beta }_{c}}{\sum_{\mathrm{c}=1}^{N}{\mathrm{Num}}_{{z}_{ij}}^{c}+{\beta }_{c}}$$

5 $${\widehat{\theta }}_{{z}_{ij}}^{{d}_{i}}=\frac{{\mathrm{Num}}_{{z}_{ij}}^{{d}_{i}}+{\alpha }_{{z}_{ij}}}{\sum_{{\mathrm{z}}_{\mathrm{ij}}=1}^{\mathrm{K}}{\mathrm{Num}}_{{z}_{ij}}^{{d}_{i}}+{\alpha }_{{z}_{ij}}}$$

In (4) and (5), c is the tag word and ${\widehat{\boldsymbol{\varphi }}}_{{{\varvec{z}}}_{{\varvec{i}}{\varvec{j}}}}^{{\varvec{c}}}$ is the distribution probability that c belongs to the topic ${{\varvec{z}}}_{{\varvec{i}}{\varvec{j}}}$; ${\widehat{{\varvec{\uptheta}}}}_{{\mathbf{z}}_{\mathbf{i}\mathbf{j}}}^{{\mathbf{d}}_{\mathbf{i}}}$ is the distribution probability that document ${{\varvec{d}}}_{{\varvec{i}}}$ contains the topic ${{\varvec{z}}}_{{\varvec{i}}{\varvec{j}}}$. Obviously, when the marked word c is a high-frequency word, the probability that it belongs to the topic ${{\varvec{z}}}_{{\varvec{i}}{\varvec{j}}}$ and the document ${{\varvec{d}}}_{{\varvec{i}}}$ is higher, resulting in the topic distribution tending to tilt toward high-frequency words. After the improvements introduced via (1) and (2), when the tag word c is assigned to the topic ${{\varvec{z}}}_{{\varvec{i}}{\varvec{j}}}$, it is given a corresponding weight. In (4) and (5), the values of ${\mathbf{N}\mathbf{u}\mathbf{m}}_{{{\varvec{z}}}_{{\varvec{i}}{\varvec{j}}}}^{{\varvec{c}}}$ and ${\mathbf{N}\mathbf{u}\mathbf{m}}_{{{\varvec{z}}}_{{\varvec{i}}{\varvec{j}}}}^{{{\varvec{d}}}_{{\varvec{i}}}}$ are given differentiated word weights ${\mathbf{W}\mathbf{e}\mathbf{i}\mathbf{g}\mathbf{h}\mathbf{t}}_{{{\varvec{w}}}_{{\varvec{i}}{\varvec{j}}}}$. The improved LDA topic model can therefore achieve a finer-grained and more reasonable division of text data by topic.

### ARMA time series model

The autoregressive moving average (ARMA) time series model is composed of an autoregressive model (AR) and a moving average model (MA) [21]. In a time series $X=\{{X}_{1},{X}_{2},\dots ,{X}_{T}\}$, the value at a certain moment in the preset time series of the ARMA model is related to the value of the first p time series and the first q random disturbances entering the system, and from this we can predict the value at the next moment. Let ${{\varvec{X}}}_{{\varvec{t}}}$ be an autoregressive process that is affected by the values of the first p time series, as shown in (6):

6 $${X}_{t}={\eta }_{1}{X}_{t-1}+{\eta }_{2}{X}_{t-2}+\dots +{\eta }_{p}{X}_{t-p}+{e}_{t,}$$

where ${{\varvec{\eta}}}_{1},{{\varvec{\eta}}}_{2},\dots ,\boldsymbol{ }{{\varvec{\eta}}}_{{\varvec{p}}}$ are autoregressive coefficients, and ${{\varvec{e}}}_{{\varvec{t}}}$ is the error term. The error term ${{\varvec{e}}}_{{\varvec{t}}}$ has a dependency on the different values of the time series, and its moving average process can be expressed in (7) as follows:

7 $${e}_{t}={\mu }_{1}{\varepsilon }_{t-1}+{\mu }_{2}{\varepsilon }_{t-2}+\dots +{\mu }_{q}{\varepsilon }_{t-q}+{\varepsilon }_{t},$$

where ${\upmu }_{1},{\upmu }_{2},\dots , {\upmu }_{\mathrm{q}}$ are moving average coefficients and ${\upvarepsilon }_{\mathrm{t}}$ is a white noise sequence. By substituting (7) into (6), the mathematical expression for the ARMA model is obtained as follows:

8 $${X}_{t}=\sum_{m=1}^{p}{\eta }_{m}{X}_{t-m}+\sum_{n=1}^{q}{\mu }_{n}{\varepsilon }_{t-n}+{\varepsilon }_{t}$$

The steps of our prediction algorithm, which is based on the ARMA model, can be described as follows:

Step 1. Zero-average processing is performed on the time series value ${{\varvec{X}}}_{{\varvec{t}}}$, and a stationarity test is then applied to ${{\varvec{X}}}_{{\varvec{t}}}$ [22]. If the result is not stable, the difference method (DM) is applied until the data after the difference are stable.

Step 2. A white noise test is carried out on the stationary data. If the test result is a stationary white noise sequence, the orders ***p*** and ***q*** of the ARMA model are obtained by calculating the autocorrelation function (ACF) and partial autocorrelation function (PACF), and a fit to the ARMA(*p*, *q*) model is calculated using the StatsModels package. The value of the Akaike information criterion (AIC) is calculated for different combinations of (*p*, *q*), and the minimum value of AIC (*p*, *q*) is taken as the estimate of (*p*, *q*).

Step 3. The least squares estimation method is used to calculate the remaining unknown parameters* η* and ***μ*** in the model. The result of dynamic prediction at instant ${\varvec{t}}+1$ is expressed as follows:

9 $${X}_{t+1}^{{^{\prime}}}={\eta }_{1}{X}_{t}^{{^{\prime}}}+\dots +{\eta }_{p}{X}_{t+1-p}^{{^{\prime}}}+{e}_{t}-{\mu }_{1}{\varepsilon }_{t}-\dots -{\mu }_{q}{\varepsilon }_{t+1-,}$$

where ${{\varvec{X}}}_{{\varvec{t}}}^{{^{\prime}}}$ is a sequence of zero mean value. In this way, the time series ARMA model is obtained to realise the predictive analysis of text data and the evolution of public opinion.

### Improved LDA-ARMA model and algorithm for sentiment analysis and prediction

**Fig. 2:**
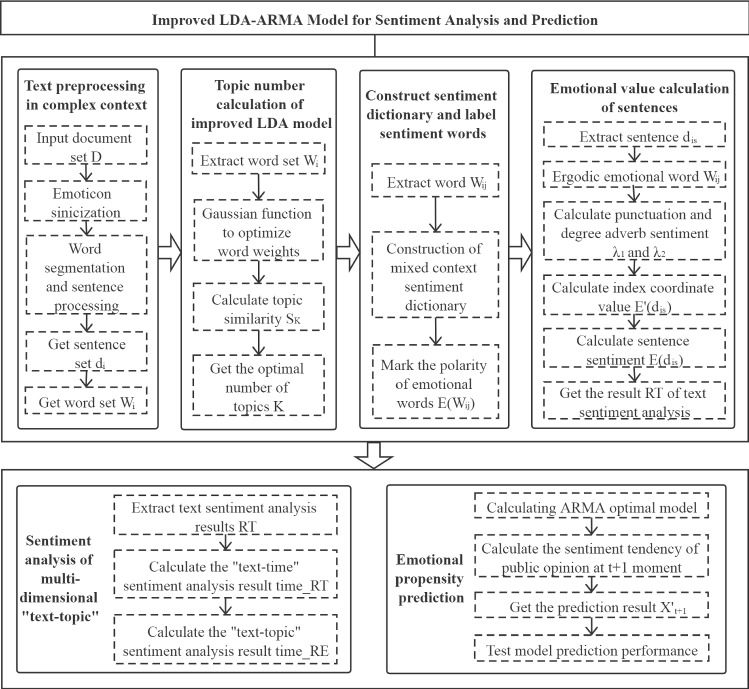
Flow chart of improved LDA-ARMA model for sentiment analysis and prediction

In order to analyse the topics in microblog text data representing public opinion of an epidemic event in a complex context, including calculation, classification and prediction of sentiment values, we propose an improved LDA-ARMA model and algorithm. A flow chart for our model is shown in Fig. 2.

The proposed model algorithm can be summarised by the following steps:

$\mathrm{Step }1.$ Pre-processing of text in a complex context. The document set ***D ***of the public opinion corpus is pre-processed by applying emoticon sinicization, English–Chinese translation, sentence segmentation, word segmentation, stop words, and punctuation marks. For each document, the sentence set ${{\varvec{d}}}_{{\varvec{i}}}$ and the word set ${{\varvec{w}}}_{{\varvec{i}}}$ are obtained.

$\mathrm{Step }2.$ Calculate the number of topics with the improved LDA model. The topic similarity is set to ${{\varvec{S}}}_{{\varvec{K}}}$, and the Gibbs sampling algorithm is used to obtain the distribution parameters for each "text topic" and "topic-word". A Gaussian function is used to adjust the weights of the high- and low-frequency words for use in (4) and (5). Finally, the similarity ${{\varvec{S}}}_{{\varvec{K}}}\boldsymbol{ }$[23] corresponding to the number of different topics K is calculated. When ${{\varvec{S}}}_{{\varvec{K}}}$ is a minimum, the number of topics K is optimal, and the improved LDA "topic word" distribution is obtained.

$\mathrm{Step }3.$ Construction of the sentiment dictionary and labelling of the sentiment words. Let ${\varvec{E}}({{\varvec{w}}}_{{\varvec{i}}{\varvec{j}}})$ be the sentiment value of the word ${{\varvec{w}}}_{{\varvec{i}}{\varvec{j}}}$. The words are matched with the constructed sentiment dictionary for the mixed-language context. The emotion polarity is automatically labelled for those words that can be automatically matched, labelling positive emotion words as ${\varvec{E}}({{\varvec{w}}}_{{\varvec{i}}{\varvec{j}}})=$ 1, negative emotion words as ${\varvec{E}}({{\varvec{w}}}_{{\varvec{i}}{\varvec{j}}})=$−1 and neutral emotion words as ${\varvec{E}}({{\varvec{w}}}_{{\varvec{i}}{\varvec{j}}})=$ 0. After sinicization, the emoticons are regarded as special emotional words ${{\varvec{w}}}_{{\varvec{i}}{\varvec{j}}}$, which are manually labelled according to the labelling rules for emotional words and are added into the emotion dictionary.

$\mathrm{Step }4.$ Calculation of the emotional value of sentences. We set the sentiment degree of a punctuation mark behind a sentiment word to ${{\varvec{\lambda}}}_{1}$, and the degree adverb in front of the emotional word as ${{\varvec{\lambda}}}_{2}$. Based on the word sentiment value ${\varvec{E}}({{\varvec{w}}}_{{\varvec{i}}{\varvec{j}}})$ obtained in Step 3, we traverse the degree adverb in front of the sentiment word. For each sentiment word ${\mathrm{w}}_{\mathrm{ij}}$, the sentiment value is calculated as ${{\varvec{\lambda}}}_{2}{\varvec{E}}({{\varvec{w}}}_{{\varvec{i}}{\varvec{j}}})$. Following this, we traverse the negative words before the emotion word; when the number of negative words is odd, the sentiment value of the sentiment word ${{\varvec{\lambda}}}_{2}{\varvec{E}}({{\varvec{w}}}_{{\varvec{i}}{\varvec{j}}})$ takes a negative value, and when the number of negative words is even or zero, a positive value is taken. We then traverse the punctuation marks behind the emotion word and match the corresponding emotion degree. If an exclamation mark appears behind the emotion word, the value of ${{\varvec{\lambda}}}_{1}$ is two; otherwise, it is one. In addition, the index coordinates of sentence ${{\varvec{d}}}_{{\varvec{i}}{\varvec{s}}}$ are labelled, and the sentiment degree ${\varvec{E}}^{\prime}\left({{\varvec{d}}}_{{\varvec{i}}{\varvec{s}}}\right)$ is calculated based on the position of the index coordinates.

10 $$E^{\prime}\left({d}_{is}\right)=\left\{\begin{array}{c}1, If {d}_{is} \,is\, the \, first\, sentence\, of\, a\, paragraph\\ 0.5, If {d}_{is} is\, the\, last\, sentence\, of\, a\, paragraph\\ 0, otherwise\end{array}\right.$$

Finally, the equation for the sentiment value ***E(***${{\varvec{d}}}_{{\varvec{i}}{\varvec{s}}}$***)*** of each sentence ${{\varvec{d}}}_{{\varvec{i}}{\varvec{s}}}$ is as follows:

11 $$E\left({d}_{is}\right)={\lambda }_{1}\sum_{j=1}^{{N}_{i}}{\lambda }_{2}E({w}_{ij})+E^{\prime}\left({d}_{is}\right)$$

Thus, the sentiment value for document ${\mathrm{d}}_{\mathrm{i}}$ is $E\left({d}_{i}\right)=\sum_{s=1}^{S}E({d}_{is})$. We then find the best threshold for positive and negative sentiment classification using the F1 score [24] and conditionally judge ${\varvec{E}}\left({{\varvec{d}}}_{{\varvec{i}}}\right)$ using a Boolean expression. When ***E(***${{\varvec{d}}}_{{\varvec{i}}}$***)*** is higher than the threshold, it is judged to be a positive emotion; otherwise, it is a negative emotion. Based on the results of this conditional judgment, we calculate the number of documents with positive sentiment E ${^{\prime}}$_pos_(${d}_{i}$), the number with negative sentiment E ${^{\prime}}$_neg_(${d}_{i}$), the mean value of documents with the positive sentiment MnE_pos_(${d}_{i}$) and the mean value of documents with the negative sentiment MnE_neg_(${d}_{i}$). The result of the document sentiment analysis is then RT = (E ${^{\prime}}$_pos_(${d}_{i}$), E ${^{\prime}}$_neg_(${d}_{i}$), MnE_pos_(${d}_{i}$), MnE_neg_(${d}_{i}$)).

$\mathrm{Step }5.$ Carry out sentiment analysis of a multi-dimensional "text topic". The results of the text sentiment analysis RT calculated in Step 4 are integrated into the time series set of documents: $\mathrm{D}\_\mathrm{time}=\left\{\left({d}_{1},{X}_{1}\right),\left({d}_{2}, {X}_{2}\right),\dots ,\left({d}_{M},{X}_{T}\right)\right\}$, based on the division into time slices, to give the result $\mathrm{time}\_\mathrm{RT}=\{{time\_RT}_{1},{time\_RT}_{2},\dots ,{time\_RT}_{T}\}$ for the emotional time series in the "text time" dimension. We then visually calculate the results for the emotional distribution under different "text-topics", and combine RT with the "topic word" distribution to obtain the emotional distribution results [25, 26]. Next, we divide $\mathrm{time}\_\mathrm{RT}$ into **K** different topic names to obtain the emotional time series distribution result time_RE = {${time\_RE}_{1}$*,*${time\_RE}_{2}$*,…,*${time\_RE}_{T}$} under different "text topic".

$\mathrm{Step }6.$ Prediction of public sentiment trend. Using time_RT as the training set of prediction model. Based on the training set, the improved LDA-ARMA model is trained iteratively until the loss function reaches a minimum and the optimal robust model is obtained [27, 28]. We use 10% of the data in the training set as the verification set and repeatedly verify the model using these data to obtain the optimal combination of hyperparameters. We then combine the training set and the verification set, and use a fivefold cross-validation method to select the optimal model to predict the public sentiment trend for time slice t + 1, and use the prediction result ${{\varvec{X}}}_{{\varvec{t}}+1}^{{^{\prime}}}$ as the test data. Finally, the prediction performance of the model is evaluated by calculating the mean absolute percentage error (MAPE) [29] for the LDA-ARMA model.

## Empirical analysis of public opinion and emotions in regard to COVID-19

### Multi-dimensional visual empirical analysis of covid-19 public opinion and emotion

To carry out an in-depth analysis of the evolution of the opinions and emotions of residents of Wuhan and other locations in China during the COVID-19 epidemic, we took COVID-19 as the research background and used “Wuhan” as the search keyword in the “Advanced Search” on Sina Weibo to crawl texts relating to the public opinion of Wuhan netizens. The position retrieval was terminated in the second stage, and the regularization method was used to exclude the microblog located in "Wuhan," and this section of the text was taken as the public opinion text of other netizens across the country. For the above conditions, we built a crawler framework using Python. From 0:00 on January 18th to 24:00 on February 10th, 2020, we collected comments from Wuhan and other Chinese netizens on Sina Weibo. In order to ensure that the data samples were as evenly distributed as possible, we collected the first 10% of the data per hour and finally got 59,579 pieces of blog data of Wuhan netizens and 67,877 pieces of data from other Internet users in China, giving a total of 127,456 items.

The text data were pre-processed following Step 1 in part C of Section II. First, the regular Python functions Findall and Subn were used to extract English words from the comment text. After machine translation, the English words were backfilled, and the emoticons in the blog posts were regularly matched and sinicised. Next, we carried out batch segmentation of the text based on Chinese words by calling the Segmentor function in the LTP word segmentation tool from the Harbin Institute of Technology. Finally, we removed invalid data and meaningless URLs. Through this iterative process, 49,187 items of data from Weibo generated by netizens of Wuhan were finally obtained, and 56,447 items from other netizens nationwide, giving a total of 105,634. The datasets were classified into a training set and a test set with a ratio of 7:3. The training and test sets for Wuhan netizens contained 34,431 and 14,756 items, respectively, and those for the other locations in China were 39,513 and 16,934, respectively. In this way, the sentence set ${\mathrm{d}}_{\mathrm{i}}$ and word set ${\mathrm{w}}_{\mathrm{i}}$ were constructed for netizens of Wuhan and other locations in China.

In order to analyse the evolution of sentiment on this topic for the two groups of residents, we used our improved LDA topic model. First, we iteratively calculated the average similarity ${\mathrm{S}}_{\mathrm{K}}$ of the number of topics using Step 2 of the algorithm, as described in part C of Sect. 2, to obtain the optimal number of topics. We carried out numerous experimental tests, setting **α** = 50/K, **β** = 0.01, and using fivefold cross-validation; the training data before and after optimisation using the Gaussian function were iterated 2000 times each, and the test data were iterated 1000 times each. When ${\mathrm{S}}_{\mathrm{K}}$ is the minimum, the number of topics *K* is the optimal value. For **K** = 4, a comparison of the experimental results shows that the topic similarity before and after application of the improved algorithm was ${{\varvec{S}}}_{{\varvec{K}}}=$ 0.737 and ${{\varvec{S}}}_{{\varvec{K}}}=$ 0.641, respectively. The LDA model improved by the use of the Gaussian function gave better performance in terms of topic clustering. The distributions of "topic words" reflecting public opinion on the COVID-19 epidemic by netizens of Wuhan and other locations in China are shown in Tables 1 and 2, respectively.

**Table 1:** Distribution of topics discussed by Wuhan netizens in regard to the COVID-19 epidemic and public opinion after application of the improved LDA algorithm

Topic	Keywords
Topic 1	Pneumonia (0.0381), Virus (0.0303), Epidemic Situation (0.0216), Mask (0.0178), Zhong Nanshan (0.0072), Diagnosis (0.0054), SARS (0.0024), Seafood (0.0013), People (0.0009)
Topic 2	Lockdown (0.0545), Wuhan (0.0351), Government (0.0314), Hospital (0.0238), Thunder God Mountain (0.0119), disinfection (0.0104), compatriots (0.0097), awesome (0.0093), no (0.0076)
Topic 3	Come on (0.0803), China (0.0674), Safety (0.0557), Zhu Yilong (0.0431), Warm Spring (0.0225), Contribution (0.0204), Relay (0.0186), Direct Broadcast (0.0176), Help (0.0164)
Topic 4	Hospital (0.0407), Help (0.0351), Worried (0.0334), Ill (0.0278), Cry (0.0195), Bed (0.0143), Quarantine (0.0142), Government (0.0096), Stay (0.0087)

**Table 2:** Distribution of topics discussed by other netizens of China in regard to the COVID-19 epidemic after application of the improved LDA algorithm

Topic	Keywords
Topic 1	Virus (0.0592), Pneumonia (0.0523), Quarantine (0.0495), Epidemic Situation (0.0462), Mask (0.0387), Responsible (0.0321), Government (0.0216), Normal (0.0178), One (0.0151)
Topic 2	Hospital (0.0643), Medical Staff (0.0435), Protect (0.0331), Infect (0.0297), Front Line (0.0285), Patient (0.0217), Angel (0.0114), Salute (0.0102), Rumor (0.0095)
Topic 3	Come on (0.0906), Safety (0.0524), Thank (0.0473), Precious (0.0364), Together (0.0143), Spring Festival (0.0127), People (0.0116), Unite (0.0104), Journalism (0.065)
Topic 4	Contribution (0.0461), Materials (0.0369), Overcome (0.0355), Red Cross (0.0314), do (0.0306), Support (0.0272), Protect (0.0224), Japan (0.0168), PUMC (0.0082)

According to the probability distribution in Table 1, the top two keywords with the highest probability in Topic 1 were *P(Pneumonia)* = 0.0381 and *P(Virus)* = 0.0303. Topic 1 is, therefore, referred to here as “Pneumonia virus”. Similarly, Topic 2 is referred to as “Lockdown of Wuhan city”, Topic 3 “Go China”, and Topic 4 “Hospital”. The topics in Table 2 are referred to as “Pneumonia virus”, “Hospital staff”, “Encourage” and “Contribution”. From the core theme words, it appears that Wuhan netizens were more concerned with the development of the epidemic situation, treatment measures and related policies, while netizens of other areas paid more attention to the overall prevention and control of COVID-19.

In order to deepen our understanding of the trend and evolution of public opinion of the COVID-19 epidemic, based on a multi-dimensional analysis of the topic, the positive and negative words of four mainstream emotion dictionaries were used to analyse microblogs. These were the HowNet Chinese vocabulary for sentiment analysis, Tsinghua University’s dictionary of praising and derogatory words in Chinese, Dalian University of Technology’s sentiment vocabulary ontology database in Chinese, and Taiwan University’s NTUSD. After filtering repeated sentiment words from the four major dictionaries, we obtained 16,639 words relating to praising words and 18,084 derogatory words. Following Step 3 of the algorithm in Sect. 1.3, emotion words relating to specific events were manually marked, and finally, 1773 new emotion words (including 647 praising words and 1126 derogatory words) were extracted. The total number of words in the emotion dictionary was 36,496.

**Fig. 3:**
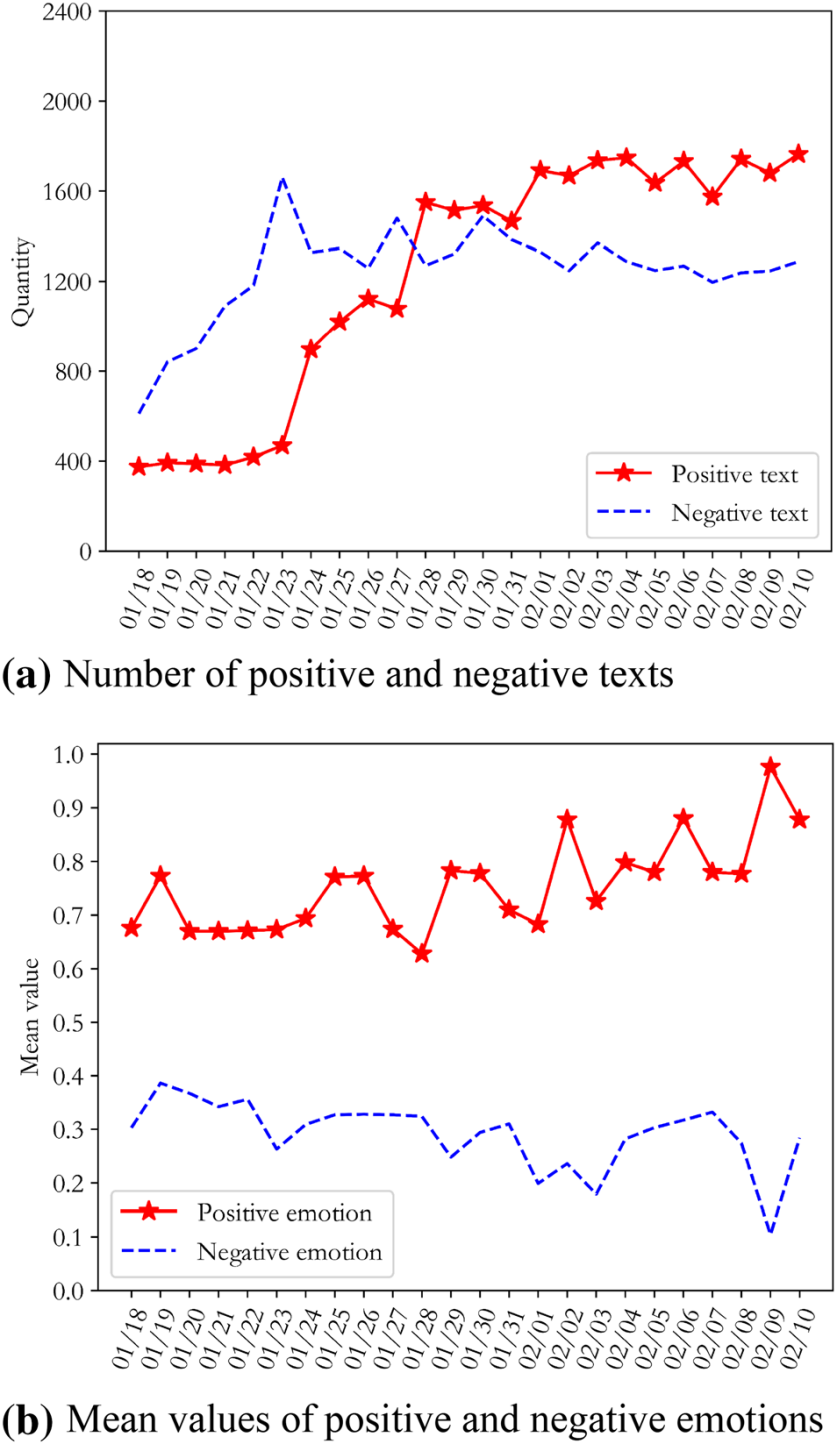
Emotional evolution and distribution of opinions of Wuhan netizens with regard to COVID-19

**Fig. 4:**
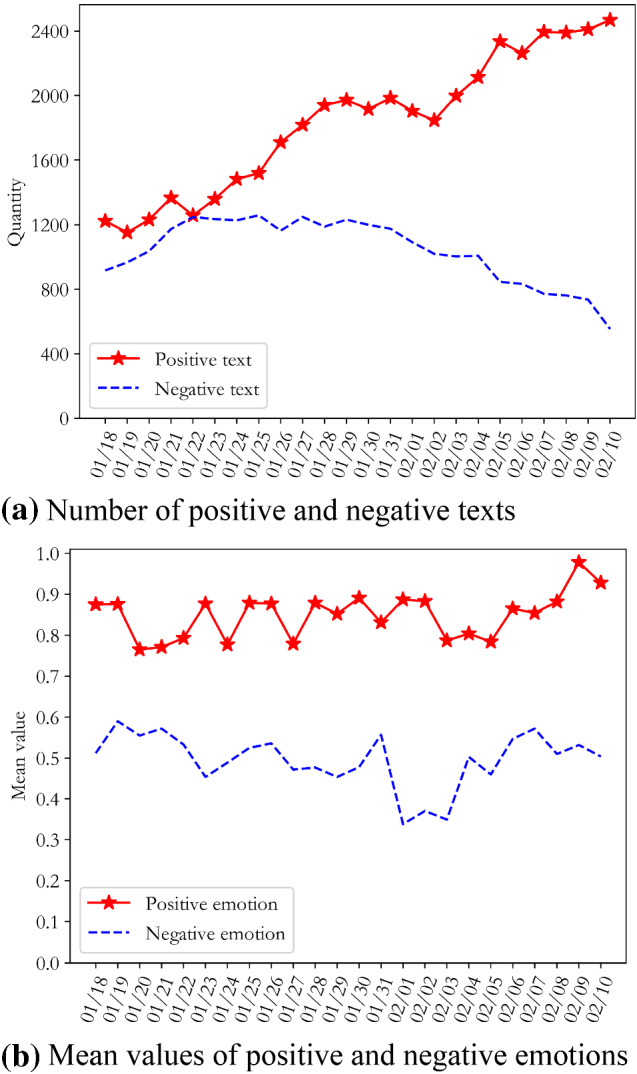
Emotional evolution and distribution of opinions of other netizens in China with regard to COVID-19

We processed these 36,496 sentiment words using Step 4 in part C of Section II, to obtain the text sentiment classification and sentiment analysis results RT(Wuhan) and RT(Nationwide). The best value for the classification threshold for positive and negative sentiments was 0.61. In order to further explore the trend in sentiment evolution from the perspective of multi-dimensional topics, according to Step 5 of the algorithm in part C of Section II, sentiment analysis was carried out on the full dataset using multiple dimensions of "text topic", and the time_RT(Wuhan) and time_RT(Nationwide) results of the "text-time" emotional time series evolution of Wuhan netizens and other netizens across the country were obtained, as shown in Figs. 3 and 4.

Figures 3a and 4a show that compared with other netizens in China, the numbers of positive and negative texts by Wuhan netizens fluctuated greatly during this period. Until 28th January, the number of negative texts by Wuhan’s netizens was always higher than the number of positive texts, reaching a peak on the 23rd January. The number of positive texts by other netizens in the country showed a steady increase during this period, and the difference between the number of positive and negative text increased. It can be inferred that residents of Wuhan, as the centre of the COVID-19 outbreak, were extremely nervous and panicked in the initial stages, due to uncertainty about the source, transmission routes, mortality rate, and treatability of the virus. Meanwhile, the lockdown of Wuhan on the 23rd January intensified the social panic and psychological pressure on Wuhan netizens. Although other netizens across the country showed panic at the beginning of the outbreak, the duration of this phase of public opinion was not long; they quickly overcame their negative emotions and turned them into positive and optimistic attitudes.

Figures 3b and 4b show that the mean values for positive emotions of Wuhan netizens and the mean values for the negative emotions of other netizens in China tended towards the optimal threshold line, indicating that the emotional attitudes of Wuhan netizens were more negative than those of other netizens in China during this period. It is worth noting that with the evolution of public opinion, the average negative sentiment of Wuhan netizens showed a downward trend overall, reflecting the fact that the outlook for the COVID-19 epidemic was not optimistic during this period.

**Fig. 5:**
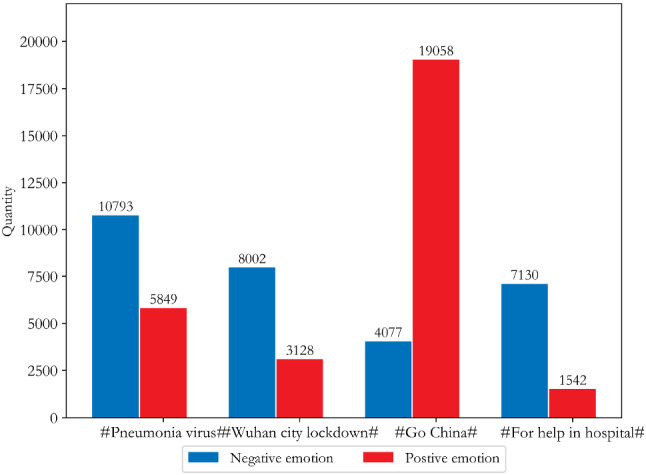
Distribution of public opinion and sentiment tendency of Wuhan netizens

**Fig. 6:**
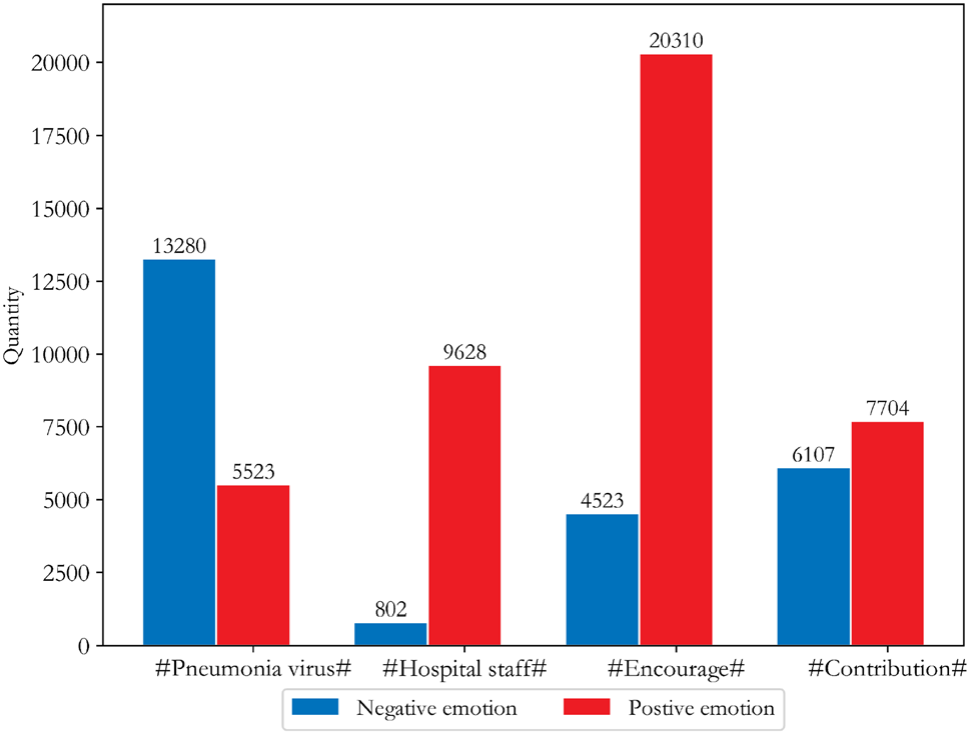
Distribution of public opinion and sentiment tendency of other netizens in China

In order to further analyse the sentiment distribution regarding the core topics in microblogs by the two groups, the "topic word" sentiment classification was combined with the coarse-grained sentiment classification result RT to obtain the "topic word" sentiment classification. The thematic sentiment distributions of netizens of Wuhan and other locations in China were obtained as shown in Figs. 5 and 6.

According to Fig. 5, except for “Go China”, Wuhan netizens had mainly negative emotions for the other three core topic words, with negative comment rates of 65%, 72%, and 82%, respectively. Wuhan netizens had more negative emotions about “Hospital” and “lockdown of Wuhan City”. Figure 6 shows that other netizens across the country had a positive and optimistic attitude towards COVID-19, especially on the themes of "Hospital staff" and “Encourage”, but the emotional attitude towards the theme of “Contribution” was mixed, and the reason for this is worth investigating further.

**Fig. 7:**
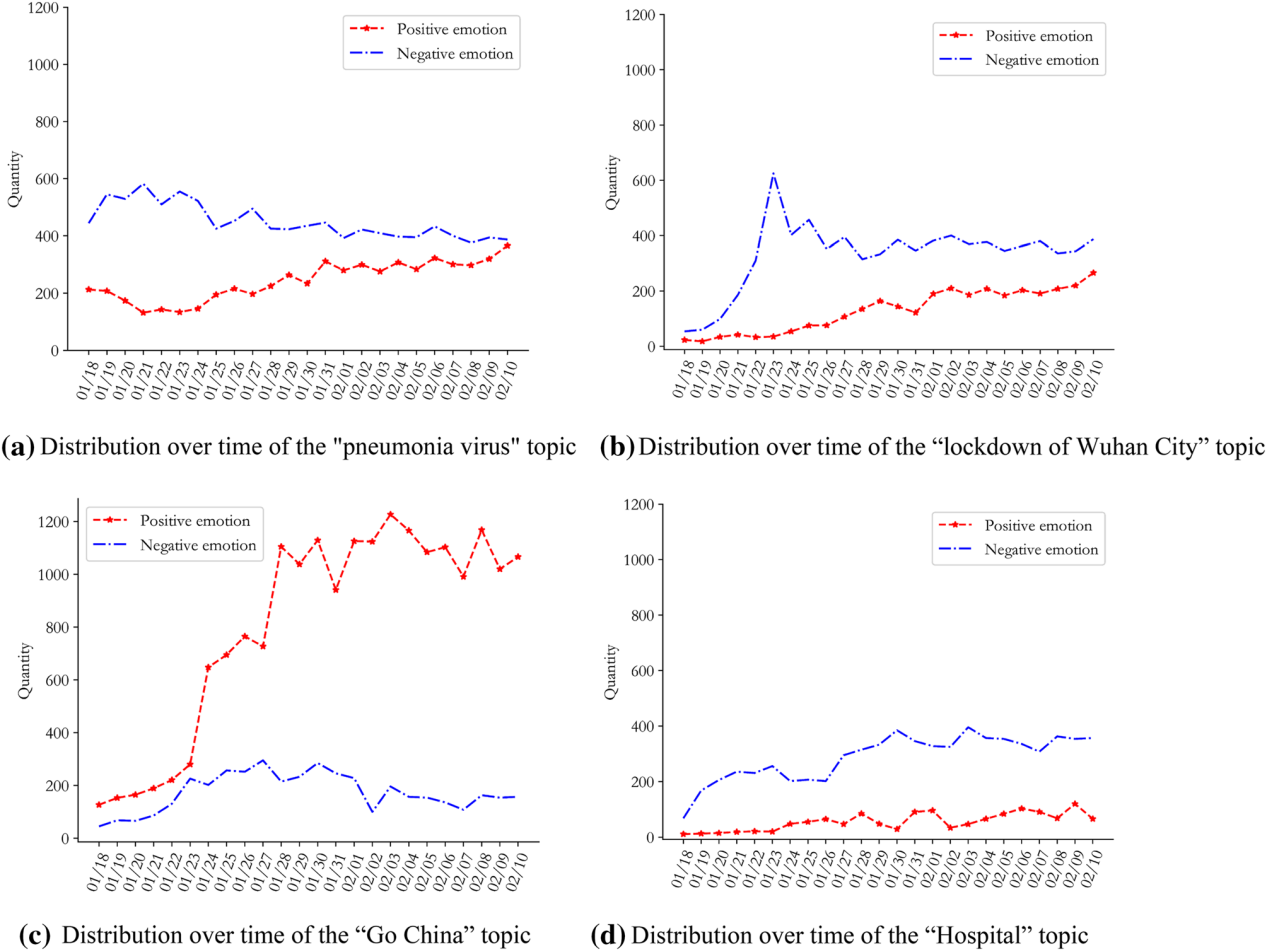
Evolution of public opinion and sentiment of Wuhan netizens on four topics of the COVID-19

**Fig. 8:**
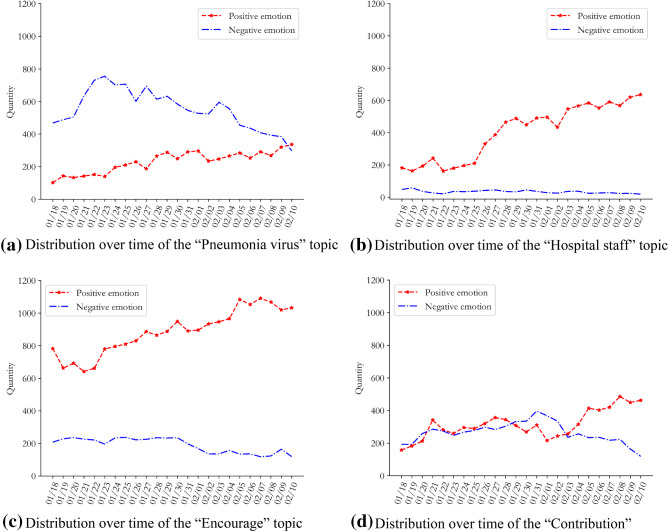
Evolution of public opinion and sentiment of other netizens in China on four topics of the COVID-19

In order to grasp the trends in the emotional evolution of public opinion on COVID-19 of the two groups over time, the distribution results for the emotional tendency of the different topics were divided into time slices (*T* = 24). We obtained the "text topic" emotional time-series distributions time_RE (Wuhan) and time_RE (Nationwide) for the two groups as shown in Figs. 7 and 8.

Figure 7 shows that for these four topics, positive emotion generally increased over time. It is worth noting that the positive text evolution paths in Figs. 3a and 7c are highly consistent. It can be inferred that the number of positive texts shown in Fig. 3a underwent rapid growth from 23rd to 28th January, mainly due to the positive emotional growth of the theme “Go China”. Although the “pneumonia virus” and “Go China” showed a slow downward trend, negative emotions related to “Hospital” continued to rise. Through tracing, it was found that most of these netizens were diagnosed patients or their families, and a small number of medical staff. It is preliminarily inferred that the medical conditions of some diagnosed patients and the issue of supplies for medical staff during this period were not adequately addressed. However, the negative emotion related to “lockdown of Wuhan City” reached its highest value in the initial stages of the crisis, and then decreased rapidly, indicating that when the news of lockdown was released, Wuhan netizens generally showed negative and panic emotions. However, with the active involvement of officials and disclosure of information, the anxiety of Wuhan netizens was alleviated and their rationality was gradually restored.

Figure 8 shows that the positive emotions for all the four topics showed an upward trend. The positive emotion related to “Hospital staff” was the largest, and the negative emotion was the smallest, indicating that netizens of other regions of China had positive emotion towards the efforts of medical staff during the COVID-19 epidemic. The highest fluctuations were seen for the negative emotions towards “donations”. Through source tracing, it was found that the negative sentiment of these netizens mainly arose from the “mask gate” incident, which stimulated negative emotions such as dissatisfaction and anger. The donation by Japan and other friendly countries to China then aroused the gratitude and praise of these residents.

Judging from the evolution of the COVID-19 public opinion of Wuhan netizens and other netizens across the country, most Wuhan netizens were actively fighting the epidemic. However, Wuhan as a disaster area, the medical resources and supplies of confirmed patients, patients' families and medical staff in Wuhan still need to be attached great importance. Other netizens across the country acted as “supervisors” for prevention and control of COVID-19 during the epidemic; they actively responded to the call for anti-epidemic measures at home and effectively reduced the probability of virus transmission through practical actions. They also actively participated in prevention and control work online, such as transmitting their experience and skills in relation to prevention and control of the disease and social circles and activity track of patients diagnosed, collecting materials or providing channels for donations to frontline workers, which provided a broad basis for the development and popularisation of epidemic prevention and control. At the same time, in view of the mistakes and anomie related to such work, the public opinion of other netizens across the country played an important role in monitoring and promoting efficient prevention and control.

### An empirical analysis of the dynamic prediction of COVID-19 public opinion and emotion

**Fig. 9:**
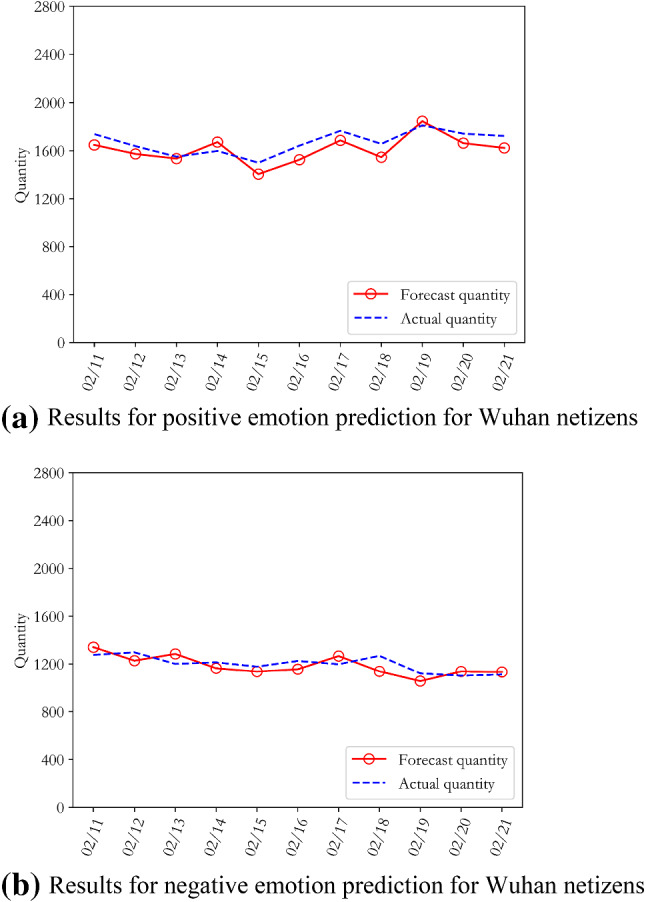
Positive and negative emotion prediction results for Wuhan netizens during the COVID-19 epidemic

**Fig. 10:**
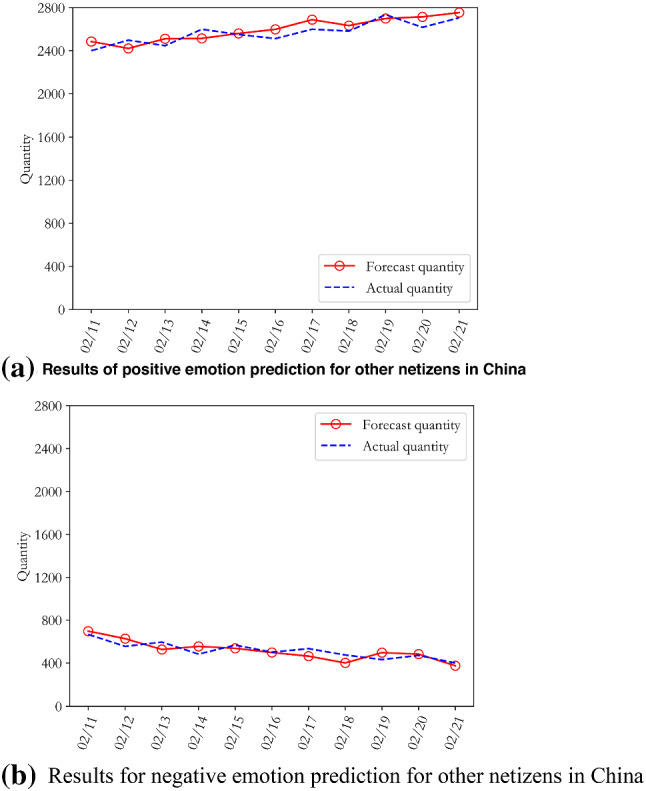
Positive and negative emotion prediction results for other netizens in China during the COVID-19 epidemic

We aimed to obtain further insight into the development trends and evolution of public opinion on COVID-19, to make accurate predictions for the development of public opinion on this issue, and to provide a reasonable theoretical basis for government departments to formulate differentiated public opinion prevention measures. Following Step 6 of the algorithm in part C of Section II, the "text-time" sentiment analysis result $\mathrm{time}\_\mathrm{RT}=\{{time\_RT}_{1},{time\_RT}_{2},\dots ,{time\_RT}_{24}\}$ was substituted into the improved LDA-ARMA hybrid model for iterative training. After cross-validating the trained optimal model on the training and verification sets, we predicted comment data and the emotional evolution process for the period from 11 to 21th February 2020. The prediction results for the positive and negative emotions of both groups of residents are shown in Figs. 9 and 10.

Figures 9 and 10 show that there was no significant change in the positive and negative emotional trends of Wuhan’s netizens in the forecast data. In general, the positive emotional trend of Wuhan netizens fluctuated, while the negative emotional trend decreased slowly. The sentiment trends of the other netizens across the country are more obvious. Compared with Wuhan netizens, the positive sentiments of these residents increased significantly, while negative sentiments decreased considerably. In fact, with the gradual decline in the diagnosis and suspected rates of new coronary penumonia, and the gradual increase in the cure rate, the negative emotions of tension and extreme panic experienced by Wuhan netizens and other netizens across the country were alleviated to varying degrees, and the themes of public opinion also became more diverse. The topics discussed by netizens began to turn to problems such as the brutal requisition of college dormitories, live broadcast chaos of school online teaching, and the emergence of COVID-19 in Shandong prison.

In order to further verify the prediction results, we calculated the average absolute percentage error MAPE values and found that the values for the positive and negative emotions of Wuhan netizens were 4.74% and 5.87%, respectively, while those of other netizens in China were 2.59% and 9.37%, respectively. It can be seen that despite the large scale of the experimental data and the presence of a mixture of languages, the MAPE values for the test set still achieved good results, with an average error rate of less than 5.64%. This proves that the improved LDA-ARMA model can effectively simulate the evolution of public opinion on COVID-19 and can predict the trends of this opinion. These results indicate that netizens of other locations in China were able to overcome negative emotions about COVID-19 more quickly, while Wuhan netizens needed more time, more powerful anti-epidemic measures and psychological counselling provided by the government departments.

## Analysis of the differences in the evolution of public opinion and sentiment between Netizens of Wuhan and other locations in China

**Fig. 11:**
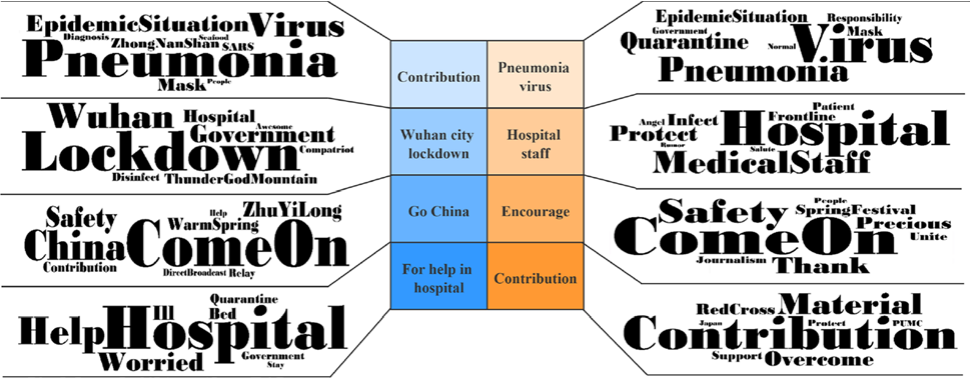
Word clouds for subjects addressed by netizens of Wuhan (left) and other locations in China (right) during the COVID-19 epidemic

In order to more intuitively reflect the differences of public opinion on this topic between residents of Wuhan and other areas of China, the topics discussed by the two groups are presented in the form of word clouds based on the probability distribution of their occurrence, as shown in Fig. 11.

From the analysis in Fig. 11 and the previous two sections, we can see that the emotional attitudes of Wuhan netizens were more negative and pessimistic than those expressed by netizens of other parts of the country. As the centre of the COVID-19 outbreak, Wuhan’s netizens lacked a deep knowledge of COVID-19 in the early stages, and this was evident from public opinion; in addition, internet media amplified the negative effects of information dissemination, and the negative emotions of Wuhan netizens spread rapidly and widely, giving rise to the threat of large-scale group psychological crises. In the early stages of the epidemic, the government of Wuhan should have taken the initiative to voice and answer questions promptly, so that information was open and transparent, thereby alleviating the negative emotions of Wuhan netizens. Second, in the middle and late stages of the epidemic, it should have provided effective psychological assistance to the masses to help them overcome the temptation to panic, which would have been conducive to social stability and development.

The emotional evolution of other netizens across the country has generally been positive and optimistic, and positive emotions have far outweighed negative ones. These netizens were relatively far away from the epidemic-stricken area, and when responding to the call for anti-epidemic disease at home, they paid a great deal of attention to the medical staff and grassroots workers who were fighting on the frontline of COVID-19. Especially when news of the heroic deeds of medical staff was widely spread via microblogs, the empathy of other netizens in China was aroused; these people were acutely aware of the difficulty of anti-epidemic work, and this resolved a certain amount of anxiety and dissatisfaction. The COVID-19 epidemic deepened the mutual understanding between doctors and patients and provided an opportunity to improve the current close relationships between doctors and patients. In addition, the central government quickly coordinated medical resources across the country, built Huoshenshan and Leishenshan hospitals, and opened numerous shelter hospitals within a short time; this provided solid reassurance in terms of the prevention and control of COVID-19, demonstrated efficient response and governance capabilities and made the public opinion of other netizens in the country develop positively spontaneously. It is undeniable that COVID-19 has greatly affected the daily life and level of production of small and medium-sized enterprises and ordinary people. In the middle and late stages of the COVID-19 epidemic, the allocation of resources by the state should be adjusted to help enterprises and individuals return to a normal (i.e. pre-epidemic) level as soon as possible.

## Conclusion

The article examines public opinion of COVID-19 as expressed online and builds an improved LDA-ARMA hybrid model for a complex context, for example involving a mixture of Chinese and English text, slang and emoticons. We analyse and predict the evolution of sentiment within public opinion data in an attempt to find the laws underlying the evolution of emotion around large-scale public opinion events and propose an algorithm to measure the emotional value of text based on machine learning. Using an evolutionary analysis of topics, this article analyses the thematic features of different research subjects in multiple dimensions, and by applying "text topic" sentiment analysis, we analyse the public opinion of netizens of Wuhan and other locations across China on different topics. We use our proposed LDA-ARMA hybrid model to predict the evolution of public opinion and emotional trends in complex contexts and show that the average error rate does not exceed 5.64%.

Based on the evolution of the emotions of Wuhan netizens, this empirical research shows that these people are under a lot of emotional pressure; there is, therefore, a need for the relevant departments to offer timely psychological assistance and guidance of public opinion to avoid a large-scale psychological crisis. The evolution trends in the emotions of other netizens in China show that their emotional states are more positive. This positive and optimistic mood can help Wuhan netizens relieve their emotional pressure, arouse the national unity and cohesion of other netizens in the country and call on the masses to actively fight COVID-19. Based on the differences in emotional evolution between netizens of Wuhan and other locations in China, the targeted allocation of the resources of the country can be achieved during the COVID-19 epidemic. In the early stages of an epidemic such as COVID-19, medical, financial and information resources should have been quickly concentrated, the disease should be stabilised quickly, and information should be open and transparent, in order to resolve the negative emotions expressed in public opinion. In the middle and later stages of the epidemic, financial and human resources should be biased towards enterprises and individuals to help them recover to pre-epidemic levels.

The limitation of this study is that it is based on single case, making it impossible to derive a general causal relationship between the level of public pleasure and government performance. This study, on the other hand, presents a preliminary framework for the government to respond rapidly to public emergencies, especially during times of public emergency. In future research, we will continue to explore the application of deep neural networks in the tracking and governance of large-scale emergencies, as well as attempt to integrate more modal data, such as video, voice and bullet screen, so as to more accurately capture the emotional evolution of Internet users, in order to provide targeted guidance and governance for Internet users in different regions or different psychological states.
